# A Case of Secondary Aortoesophageal Fistula Inserted a Covered Self-Expanding Esophageal Stent to Control Gastrointestinal Bleeding

**DOI:** 10.1155/2013/857135

**Published:** 2013-05-28

**Authors:** Makoto Onodera, Yoshihiro Inoue, Yasuhisa Fujino, Satoshi Kikuchi, Shigeatsu Endo

**Affiliations:** Iwate Medical University, School of Medicine, Department of Emergency Medicine, 19-1 Uchimaru, Morioka, Iwate 020-8505, Japan

## Abstract

A 73-year-old man presented with melena. After a thorough workup including esophageal endoscopy, computed tomography scans, and esophagography, the diagnosis of secondary aortoesophageal fistula was made. Two years previously, he had undergone endovascular stent-graft repair for the dissection of his descending thoracic aorta. Because of the generally poor condition of the patient and the high risk of any aggressive surgical intervention, we inserted a covered self-expanding esophageal stent on postadmission day 18. Esophagography after insertion did not show any evidence of a leak of contrast medium. Despite treatment with antibiotics, he developed sepsis and expired on day 52, but rebleeding did not occur in this period. We consider insertion of a covered self-expanding esophageal stent as a feasible option in the management of secondary aortoesophageal fistula in high-risk patients.

## 1. Introduction

Secondary aortoesophageal fistula (AEF) after thoracic endovascular aortic repair (TEVAR) is relatively rare [1–6], with a reported incidence of 1.7% to 1.9% [4, 7]. Treatment options are very limited, as these patients are usually not candidates for open surgery. Outcomes with conservative management are almost always fatal due to recurrent hemorrhage or chronic mediastinitis. Of note, there are no treatments to manage spontaneous, recurrent hemorrhage. In this paper, we describe a case of secondary AEF with insertion of a covered self-expanding esophageal stent to control gastrointestinal bleeding. 

## 2. Case Presentation

**Figure 1:**
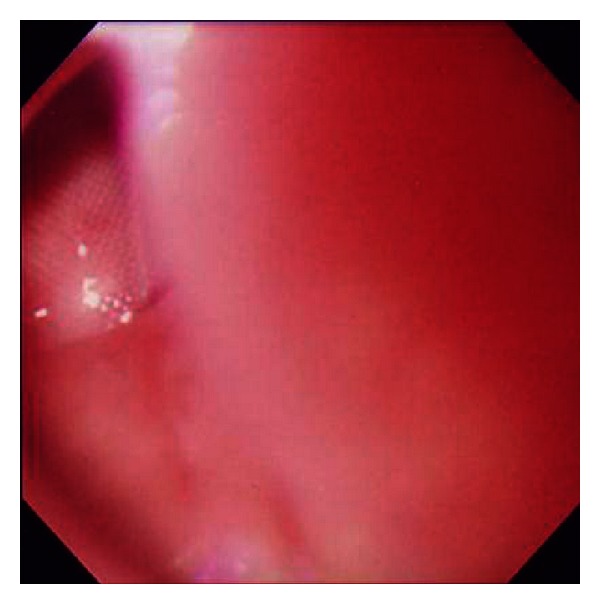
Esophageal endoscopy on hospital day five showed an esophageal ulceration with the prosthetic aortic graft visible at the base.

**Figure 2:**
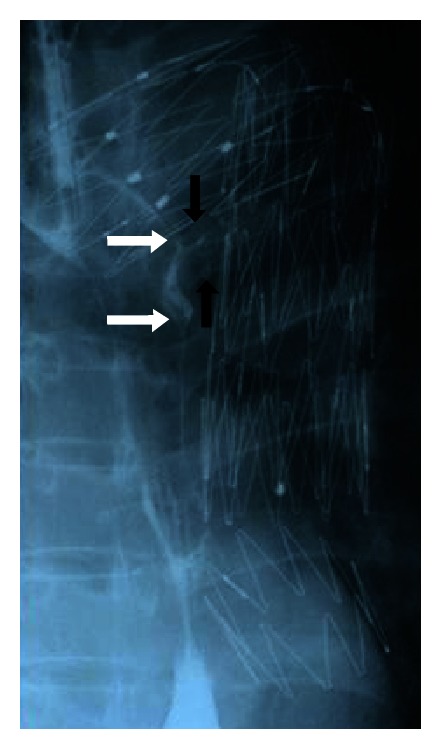
Esophagography revealed an elevated lesion (white arrows) and a fistulous tract toward the stent graft (black arrows).

**Figure 3:**
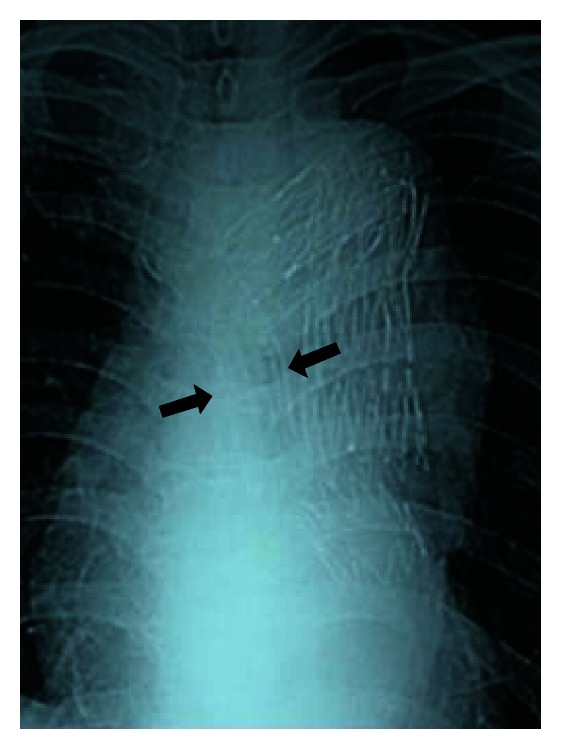
On postadmission day 18, chest X-ray revealed a covered self-expanding esophageal stent (black arrows).

A 73-year-old man presented with melena. He had a history of endovascular stent-graft repair for the dissection of a descending thoracic aorta at the age of 71 and a stent-graft repair for a pseudoaneurysm sac of the distal aortic arch at the age of 72. He was hemodynamically unstable, and the bulbar conjunctiva showed evidence of anemia at presentation. Esophago-gastro-duodenoscopy (EGD) on admission revealed a white polyp in the midesophagus but no signs of acute bleeding. Colonoscopy was unremarkable. A blood transfusion failed to raise the hematocrit. On postadmission day five, the patient had hematemesis, prompting repeat EGD. Closer inspection of the previously seen esophageal polyp showed that the white material at the base was in fact the wall of the aortic interposition graft (Figure 1). Chest computed tomography (CT) scans revealed a high-density spot in contact with the esophagus and low-density spots in the vicinity of the stent graft. Esophagography showed a fistulous tract toward the stent graft (Figure 2). We concluded that the gastrointestinal bleeding was caused by an AEF. Given the generally poor condition of the patient and the high risk of any aggressive surgical intervention, we elected to insert a covered self-expanding esophageal stent on postadmission day 18 (Figure 3). Esophagography after insertion did not show any evidence of a leak of contrast medium. Despite the use of broad-spectrum antibiotics, the patient became septic and eventually expired on postadmission day 52. Of note, however, rebleeding did not occur in this period. 

## 3. Discussion

Generally, conservative management is the mainstay of treatment of secondary AEF after TEVAR as most of these patients have contraindications to major surgery given their poor general condition [4, 6–9]. Conservative management includes the use of broad-spectrum antibiotics, medical blockade of gastric acid with a proton-pump inhibitor, and enteral feeding via percutaneous endoscopic gastrostomy (PEG) to protect the esophageal lesion [1]. However, outcomes with conservative management are almost invariably fatal due to recurrent hemorrhage or chronic mediastinitis [4, 7–9].Kasai et al. reported a case that survived with conservative management for 14 months after the initial bleeding event, a long survival period for this condition. But the fistula in this case was small, with EGD demonstrating only a small esophageal ulcer [6]. In our case, EGD and esophagography demonstrated a fistulous tract toward the stent graft. Eggebrecht observed patients with AEF for 4 weeks and found no cases of these fistulae healing. All of these patients developed fatal rebleeding [4]. Therefore, conservative management may be effective only in cases of small fistulae. 

There are few reports describing self-expanding esophageal stents to promote healing of esophageal fistulae and to prevent rebleeding. Eggebrecht et al. inserted self-expanding esophageal stents in 3 patients with secondary AEF after endovascular stent grafting, with two patients surviving for 2–6 months [4]. In our case, a covered self-expanding esophageal stent was inserted to prevent rebleeding, and rebleeding did not occur for 52 days after admission. Therefore, placement of a covered self-expanding esophageal stent is useful to prevent rebleeding for a substantial period of time. 

AEF after TEVAR may develop secondary to sepsis in cases where there is the presence of an endoleak inside the aneurysmal sac, or in cases of graft infection. In our case, the patient developed sepsis despite the use of broad-spectrum antibiotics, and despite no evidence of leak on the poststent placement esophagram. There is no consensus in the literature regarding optimal choice and length of antimicrobial therapy for an infected aneurysmal sac. Generally, antibiotics are given intravenously at the maximally tolerated dosage for at least the initial 4–6 weeks, followed by a sequential oral regimen once the acute phase of the infection has subsided [10]. Numan et al. reported a case of an infected aneurysmal sac in the setting of secondary AEF after TEVAR treated by CT-guided insertion of a drainage catheter. This method may help to divert infected material away from the fistulous tract communicating with the aorta. However, most stent-graft infections are caused by microorganisms that are highly virulent pathogens, such as methicillin-resistant *Staphylococcus aureus*, *Streptococcus* spp., or gram-negative species, such as *Pseudomonas* and *Klebsiella* [10], making infection control difficult even with the appropriate antibiotic therapy.

In conclusion, we consider the placement of a covered self-expanding esophageal stent to be useful in the management of secondary AEF after TEVAR in regards to prevention of rebleeding.
